# Pollens destroy respiratory epithelial cell anchors and drive alphaherpesvirus infection

**DOI:** 10.1038/s41598-019-41305-y

**Published:** 2019-03-18

**Authors:** Jolien Van Cleemput, Katrien C. K. Poelaert, Kathlyn Laval, Francis Impens, Wim Van den Broeck, Kris Gevaert, Hans. J. Nauwynck

**Affiliations:** 1 Department of Virology, Parasitology and Immunology, Faculty of Veterinary Medicine, Ghent University, Salisburylaan 133, 9820 Merelbeke, Belgium; 2 Department of Molecular Biology, Princeton University, 119 Lewis Thomas Laboratory, Washington Road, Princeton, New Jersey 08544 USA; 3 VIB Center for Medical Biotechnology, Albert Baertsoenkaai 3, 9000 Ghent, Belgium; 4 VIB Proteomics Core, Albert Baertsoenkaai 3, 9000 Ghent, Belgium; 5 Department of Biomolecular Medicine, Ghent University, Albert Baertsoenkaai 3, 9000 Ghent, Belgium; 6 Department of Morphology, Faculty of Veterinary Medicine, Ghent University, Salisburylaan 133, 9820 Merelbeke, Belgium

## Abstract

Pollens are well-known triggers of respiratory allergies and asthma. The pollen burden in today’s ambient air is constantly increasing due to rising climate change and air pollution. How pollens interact with the respiratory mucosa remains largely unknown due to a lack of representative model systems. We here demonstrate how pollen proteases of Kentucky bluegrass, white birch and hazel selectively destroy integrity and anchorage of columnar respiratory epithelial cells, but not of basal cells, in both *ex vivo* respiratory mucosal explants and *in vitro* primary equine respiratory epithelial cells (EREC). In turn, this pollen protease-induced damage to respiratory epithelial cell anchorage resulted in increased infection by the host-specific and ancestral alphaherpesvirus equine herpesvirus type 1 (EHV1). Pollen proteases of all three plant species were characterized by zymography and those of white birch were fully identified for the first time as serine proteases of the subtilase family and meiotic prophase aminopeptidase 1 using mass spectrometry-based proteomics. Together, our findings demonstrate that pollen proteases selectively and irreversibly damage integrity and anchorage of columnar respiratory epithelial cells. In turn, alphaherpesviruses benefit from this partial loss-of-barrier function, resulting in increased infection of the respiratory epithelium.

## Introduction

Asthma and seasonal rhinitis are two allergic diseases with increasing morbidities worldwide^1,2^. Plant pollens are well-known triggers of respiratory allergies and their importance is currently rising due to today’s modern society^3,4^. Indeed, global warming and today’s air pollution already led to longer-lasting and increased pollen concentrations in the ambient air and might cause future massive burdens if not prevented. How pollens interact with the respiratory mucosa remains largely unknown due to a lack of representative model systems.

It is generally believed that upon inhalation by humans or animals, pollens liberate a plethora of substances by hydration in the respiratory tract, including allergens and proteases. These pollen proteases might facilitate the para-cellular transport of pollen allergens by impairing the epithelial barrier^3,5–7^. The epithelial barrier is normally preserved by firm intercellular junctions (ICJ), which create a network of close connections between adjacent cells and maintain epithelial integrity. Next, the delivery of pollen allergens to sub-epithelial antigen presenting cells initiates the priming of T helper 2 (Th2) cells, a key step in the immunopathology of allergy^8^. This hypothesis is based on previous studies showing that pollen proteases are able to disrupt epithelial integrity in continuous cell lines^6,7,9^. More precisely, these studies demonstrated that pollen proteases disrupt major constituents of intercellular junctions (ICJ), namely occludin, zonula occludens-1 protein, claudin-1 and E-cadherin. In contrast, a more recent study using better representative primary isolated human bronchial epithelial cells showed that the epithelial barrier is not disrupted upon treatment with pollen diffusates^10^. The discrepancy between these studies could be explained by the model systems used. For instance, genetic mutations in continuous cell lines may have altered cellular phenotypes and mechanisms, leading to inconclusive or erroneous results when using these cells^11^. In addition, continuous cell lines cannot fully mimic the *in vivo* 3D architecture. Primary isolated cells represent a more valuable tool to study merely epithelial characteristics such as integrity and polarity and mimic the *in vivo* airway epithelium very well, consisting of a heterogeneous population of ciliated cells, basal cells and (mucus-)secreting cells^12–14^. However, merely epithelial cells do not fully represent a complete respiratory mucosa, which is build-up of a ciliated pseudostratified epithelium, basement membrane and underlying supportive connective tissue embedded with a repertoire of immune cells. Explants provide a good alternative to the previously mentioned models, as they maintain the *in vivo* 3D micro-environment, including all layers of the respiratory mucosa. In addition, several explants can be obtained from one animal to test multiple conditions, limiting the number of experimental animals and inter-animal variations^15–17^. Unfortunately, human *ex vivo* models are sparse and there is no clear vision on the exact impact of pollen proteases on the respiratory epithelium so far. Here, the horse (*Equus caballus*) was used as our study system. Similarly to human pollen allergies, horses commonly suffer from summer pasture-associated recurrent airway obstruction (SP-RAO) during late winter-spring, as a result of peaking pollen concentrations in the ambient air^18^. In addition, the horse has already been shown to act as a representative model to study allergies^19^.

Pollen proteases are mainly localized at the pollen wall and assist in the assembly of a pollen tube within the flower pistil’s transmission tract to deliver the male gametophyte to its female counterpart^20,21^. They have already been characterized as serine, cysteine, threonine and metalloproteinase in several plants including maize, white birch, pine, giant ragweed, rye and blue grass, easter lily and olive tree^6,7,22–24^. However, limited knowledge on plant genomics and proteomics hindered previous researchers from fully identifying these proteases.

Further, we hypothesized that a plausible pollen protease-induced disruption of epithelial barrier function might subsequently favour pathogen invasion. For instance, alphaherpesviruses often colonize new hosts through the respiratory mucosae, but primary infections are usually restricted in healthy hosts^25–28^. We recently showed that this restriction in viral replication is due to the fact that a main alphaherpesvirus receptor is located basolaterally and only becomes accessible upon artificial disruption of the epithelial barrier^29^. Hence, pollen proteases might drive alphaherpesvirus infection by exposing their main binding receptor upon disruption of epithelial integrity. The horse-specific equine herpesvirus type 1 (EHV1) is a well-known member of the alphaherpesvirus family and is closely related to other alphaherpesviruses (e.g. varicella-zoster virus, herpes simplex virus, pseudorabies and bovine herpesvirus type 1)^30,31^. Interestingly, EHV1 even stood out as the most central one among all examined alphaherpesviruses^30^. Thus, pollen-induced effects and host-specific alphaherpesvirus infections can optimally be studied using representative equine *ex vivo* and *in vitro* models, known to mimic *in vivo* conditions^12,16^.

Together, our study aimed at identifying specific plant proteases and depicting their impact on the respiratory epithelium and on subsequent alphaherpesvirus host invasion, using representative models.

## Results

### Pollen grains of Kentucky bluegrass (KBG), white birch (WB) and hazel (H) release proteases with major metalloproteinase and serine peptidase activities

**Figure 1:**
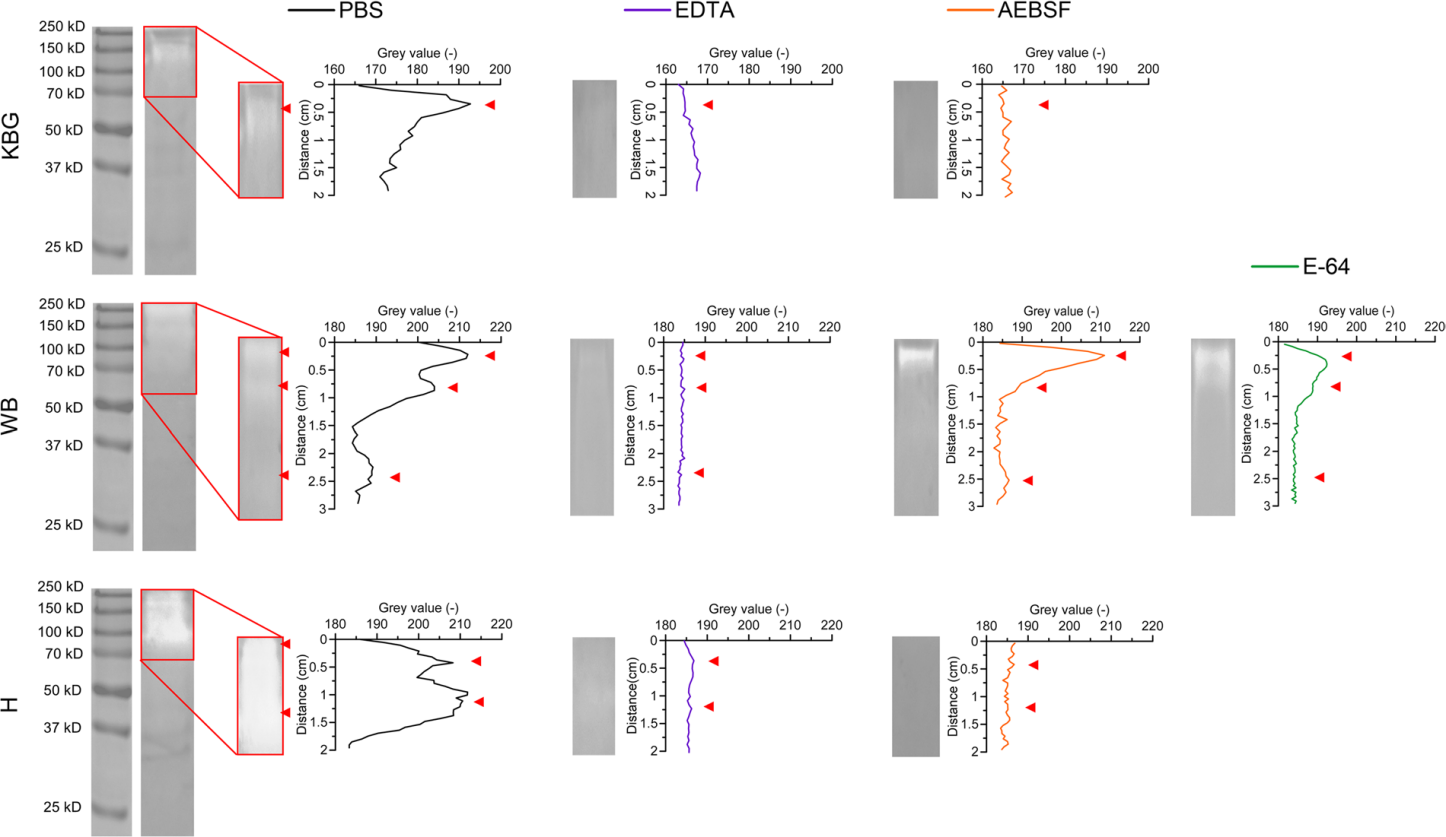
Pollen diffusates of Kentucky bluegrass (KBG; upper panel), white birch (WB; middle panel) and hazel (H; lower panel) contain proteolytically active compounds. Pollen diffusates were incubated with PBS or several protease inhibitors (2.5 µM EDTA, 500 µM AEBSF or 15 µM E-64) prior to and during zymography on a gelatin substrate. Following overnight digestion at 37 °C, Coomassie blue staining of the gelatin gels was performed. RGB-pictures were taken with a ChemiDocMP Imaging System (Bio-Rad) and converted to 8-bit grey-scaled images using ImageJ. A complete overview of the zymogram lanes of PBS-treated pollen diffusates is shown on the left (cropped images). The red box designates the area that was used for plot profiling in ImageJ. Plot profiles and their corresponding zymogram lanes are shown in the right set of graphs (cropped images). Proteolytic bands appear as white zones in the Coomassie blue-stained gelatin gel and are, together with their corresponding grey value peaks, designated by red arrowheads. Samples were derived from the same pollen diffusate batches and zymograms were processed in parallel. Original zymograms are given in Fig. S1.

Proteolytic activities of the pollen diffusates were first determined by gelatine zymography using specific protease inhibitors (PI) (Fig. 1). Plot profiling of pretreated pollen diffusate lanes was performed by means of image analysis to determine the absence or presence of specific proteolytic bands. Following control treatment with PBS, all three pollen diffusates contained proteolytic bands with motilities ranging from 70 kDa to over 250 kDa. In the KBG, WB and H lanes, one, three and two major proteolytic bands were identified, respectively. Sequestration of metal ions by EDTA inhibited metalloproteinases as well as proteases that require metal ions for stabilisation and/or activation. This was sufficient to block merely all proteolytic bands in the three pollen diffusates, as designated by a drop in the respective plot profiles. Treatment of all three pollen diffusates with the serine protease inhibitor AEBSF clearly inhibited enzymatic activities in all proteolytic bands, except for two proteolytic bands in the WB lane, located slightly above 250 kDa and at 70 kDa. The former band was only efficiently blocked following sequestration of metal ions by EDTA, suggesting that it harboured a metalloproteinase, while the latter band disappeared after inhibition of cysteine protease activity with E-64. Inhibiting aspartyl proteases with pepstatin A was not sufficient to block the proteolysis of gelatine in the proteolytic lanes of all pollen diffusates. Together, our zymography data indicate the presence of significant protease activity in plant pollen diffusates.

### Protease identification in the pollen diffusate of white birch

**Figure 2:**
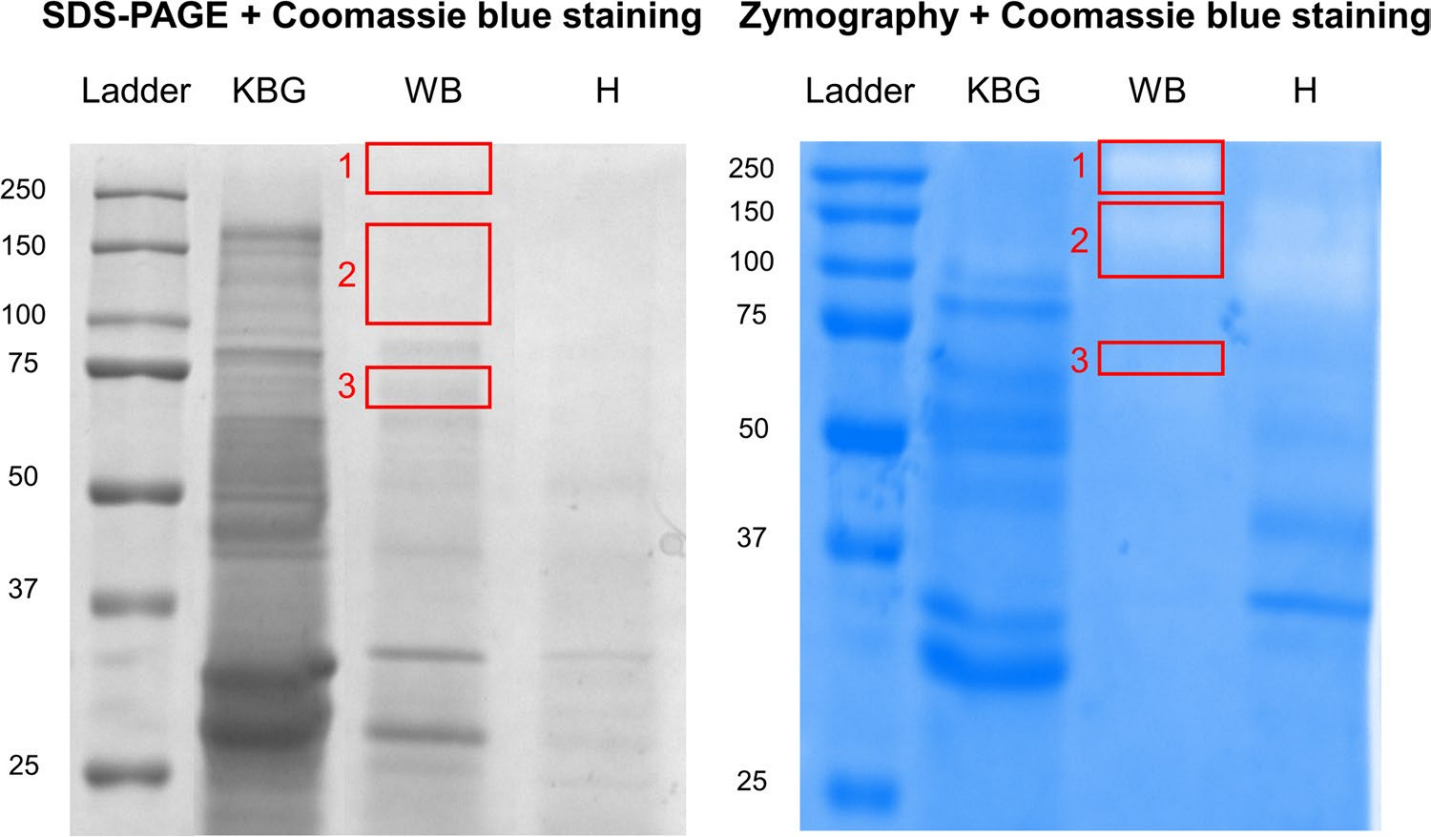
Protein (left) and proteolytic (right) profiles of pollen diffusates of Kentucky bluegrass (KBG), white birch (WB) and hazel (H). Three bands (1–3, left red boxes), corresponding to major proteolytic spots on zymography (1–3, right red boxes), were excised in the WB lane following SDS-PAGE and were subjected to LC-MS/MS analysis for protein identification.

The recent genomic sequencing of WB and accompanying annotation of its putative protein sequences^32^ enabled us to reveal the identity of WB proteases using mass spectrometry (MS)-based proteomics. For this purpose, LC-MS/MS analysis was performed on three protein bands corresponding to different enzymatic bands on the zymograms (i.e. mobility at >250 kDa [1], 100–150 kDa [2] and 70 kDa [3]), which were excised following regular SDS-PAGE (Fig. 2). The raw MS/MS data were searched against a database of *Betula pendula* proteins for which functional annotation is available from orthologous proteins in *Arabidopsis thaliana*, a widely used model in molecular plant biology and an overview of identified proteases, glycosidases and lipases is given in Table 1. Further information on the *Arabidopsis thaliana* orthologous proteases can be found using their MEROPS accession number. MEROPS is a peptidase database which groups proteases of similar evolutionary origins into (sub)clans and (sub)families. In accordance with our zymography results, primarily serine proteases were identified in all three protein bands. In particular, the ‘subtilisin-like protease SBT1.7′ (Bpev01.c0015.g0096.m0001) was predominantly recovered from all three lanes, indicating that it might exist in multiple forms. The identification of two additional subtilisin-like serine proteases (i.e. Bpev01.c1354.g0003.m0001 or ‘subtilisin-like protease SBT5.4′ and Bpev01.c0577.g0026.m0001 or ‘subtilisin-like serine protease’) in two out of three protein bands depicts a major presence of subtilases in the WB pollen diffusate. In addition, meiotic prophase aminopeptidase 1 (Bpev01.c0170.g0046.m0001), a metalloproteinase with aminopeptidase activity was identified in the protein band with a size corresponding to 100–150 kDa. Besides these proteases, a repertoire of glycosidases and lipases were also found in different WB lanes (Table 1).

**Table 1:** Identification of white birch proteases, glycosidases and lipases.

Type (and MEROPS accession)	Protein ID	Corresponding Arabidopsis gene	Description	Lane (kD)	Sequence coverage %	Nr. of unique peptides
Serine protease (S08.112)	Bpev01.c0015.g0096.m0001	AT5G67360	Subtilisin-like protease SBT1.7	>250	3.3	2
				100–150	14.6	10
				70	3	2
Serine protease (S08.A26)	Bpev01.c1354.g0003.m0001	AT5G59810	Subtilisin-like protease SBT5.4	100–150	1.2	1
				70	1.2	1
Serine protease (S08.A22)	Bpev01.c0577.g0026.m0001	AT1G20160	Subtilisin-like serine protease	100–150	1.8	1
Metalloproteinase, aminopeptidase (M01.029)	Bpev01.c0170.g0046.m0001	AT1G63770	Meiotic prophase aminopeptidase 1	100–150	1.5	1
Glycosidase	Bpev01.c0504.g0008.m0001	AT4G20050	Polygalacturonase QRT3	>250	8.4	4
				100–150	33.1	18
Glycosidase	Bpev01.c0298.g0032.m0001	AT3G48950	Endo-polygalacturonase-like protein	70	3.1	2
Glycosidase	Bpev01.c1877.g0001.m0001	AT5G64570	Beta-d-xylosidase	>250	1.8	1
				100–150	5.8	4
Glycosidase	Bpev01.c0313.g0001.m0001	AT5G04885	Glycosyl hydrolase family protein	>250	1.5	1
				100–150	6.3	1
Glycosidase	Bpev01.c0313.g0002.m0001	AT5G20950	Glycosyl hydrolase family protein	100–150	1.4	1
				70	3.3	1
Glycosidase	Bpev01.c0031.g0026.m0001	AT5G12950	Putative glycosyl hydrolase of unknown function (DUF1680)	70	3.1	3
Lipase	Bpev01.c0547.g0017.m0001	AT4G13550	Triglyceride lipase	>250	1.7	1
				100–150	3.2	2
				70	4.1	3

Proteases, glycosidases and lipases identified via proteome analysis of three protein bands (>250 kDa [1], 100–150 kDa [2], and 70 kDa [3]) from the white birch pollen diffusate, corresponding to proteolytic spots on zymography (shown in Fig. 2).

### Pollen proteases selectively and irreversibly alter integrity and anchorage of columnar respiratory epithelial cells

Pollen proteases of KBG and WB have been shown to affect epithelial integrity in Madin-Darby canine kidney (MDCK) cells and in human lung adenocarcinoma Calu-3 cells^6,7^. However, these continuous cell lines do not reflect the *in vivo* situation. Therefore, we examined the effect of pollen proteases on representative *ex vivo* respiratory mucosal explants and primary equine respiratory epithelial cells (EREC).

### Respiratory mucosal explants

**Figure 3:**
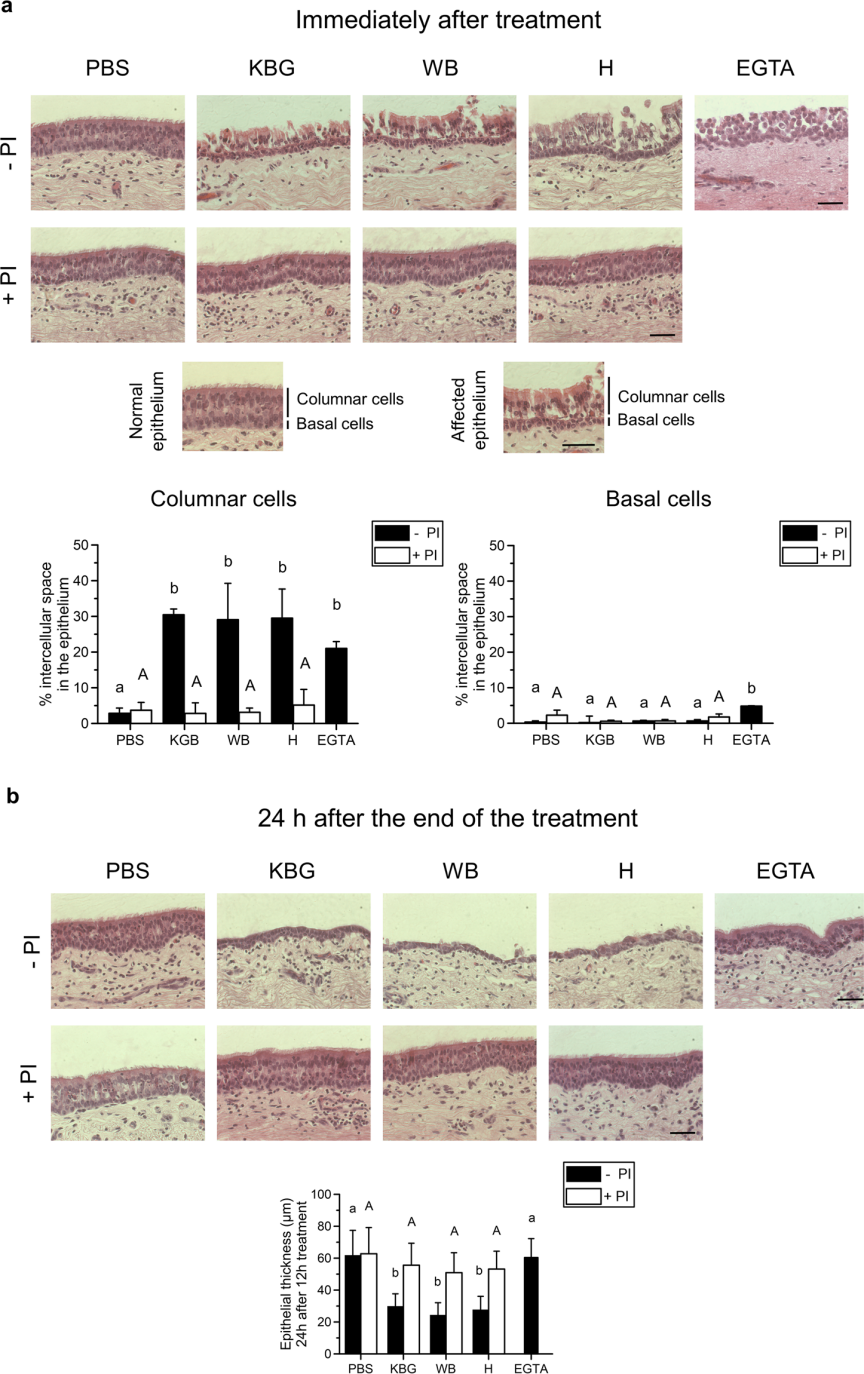
Pollen proteases selectively and irreversibly alter intercellular junctions of columnar equine respiratory epithelial cells in respiratory mucosal explants. Explants were treated with PBS (control), pollen diffusates (Kentucky bluegrass or KBG, white birch or WB and hazel or H) supplemented with or without protease inhibitors (PI) for 12 h or EGTA for 1 h. (**a**) Representative haematoxylin-eosin-stained images of the explants after treatment. The scale bar measures 50 µm (upper panel). Schematic representation of the columnar and basal layer of normal and pollen protease-affected respiratory epithelia (middle panel). The percentage of intercellular spaces in the columnar (left graph) and basal (right graph) cell layer of the respiratory epithelium after treatment (lower panels). The percentage of intercellular space was determined using ImageJ software. More precisely, the region of interest (ROI, i.e. the columnar epithelial layer or basal epithelial layer) was drawn manually for each picture in the “ROI manager tool”. Next, the threshold value to distinguish blank spaces from cellular material was determined and the percentage of blank spaces between the cells (i.e. the intercellular space) was calculated. Experiments were performed on explants from 3 individual horses and data are represented as means + SD. Different lower case letters indicate significant (P < 0.05) differences in treatments without PI, different upper case letters indicate significant (P < 0.05) differences in treatments with PI. (**b**) Representative haematoxylin-eosin-stained images of the explants 24 h after the 12 h (pollen diffusates) or 1 h (EGTA) treatment. The scale bar measures 50 µm (upper panel). The height of the epithelium was measured 24 h after treatment (lower panel). Experiments were performed on explants from 3 individual horses and data are represented as means + SD. The lower case letters indicate significant (P < 0.05) differences in treatments without PI, different upper case letters indicate significant (P < 0.05) differences in treatments with PI.

As shown in the haematoxylin-eosin-stained images of Fig. 3a, the intercellular spaces opened up following treatment with all three pollen diffusates, compared to control treatment with PBS. There was no effect of any of the three pollen diffusates on epithelial integrity when protease inhibitors (PI) were added (500 µM AEBSF, 15 µM E-64 and 2.5 µM EDTA), demonstrating that pollen proteases affect epithelial intercellular junctions (ICJ). Notably, basal cell ICJ were resistant to treatment with pollen proteases throughout the course of the experiment, as shown by the intact basal cell layer on haematoxylin-eosin-stained sections. Indeed, the intercellular space significantly (P < 0.05) increased in the columnar cell layer (left graph), but not in the basal cell layer (right graph). As a control, treatment with the Ca^2+^-chelating agent EGTA efficiently disrupted the ICJ of both basal and columnar cells, as demonstrated by the significant increase (P < 0.05) in intercellular space in both cell layers. Viability of cells within the respiratory epithelium did not significantly drop after treatment with pollen diffusates, compared to control PBS (Fig. S2a, left panel). However, desquamating cells did show signs of apoptosis, as shown in the right panel of Fig. S2a (green signal).

In contrast to EGTA, the pollen diffusates irreversibly affected epithelial morphology. Twenty-four hours after the 12 h pollen diffusate treatment, the height of the epithelium was significantly lower (KBG, 30 ± 8 µm; WB, 24 ± 8 µm; H, 28 ± 8 µm), compared to PBS or EGTA treatment (62 ± 15 µm and 60 ± 12 µm, respectively), as shown in Fig. 3b. In addition, the ciliated appearance of the respiratory epithelium was lost upon incubation with the pollen diffusates. Again, these effects were attributed to the protease activities of all three pollen diffusates, as the addition of PI prevented these changes in epithelial morphology. Representative haematoxylin-eosin-images are given in the upper panel of Fig. 3b.

#### EREC

**Figure 4:**
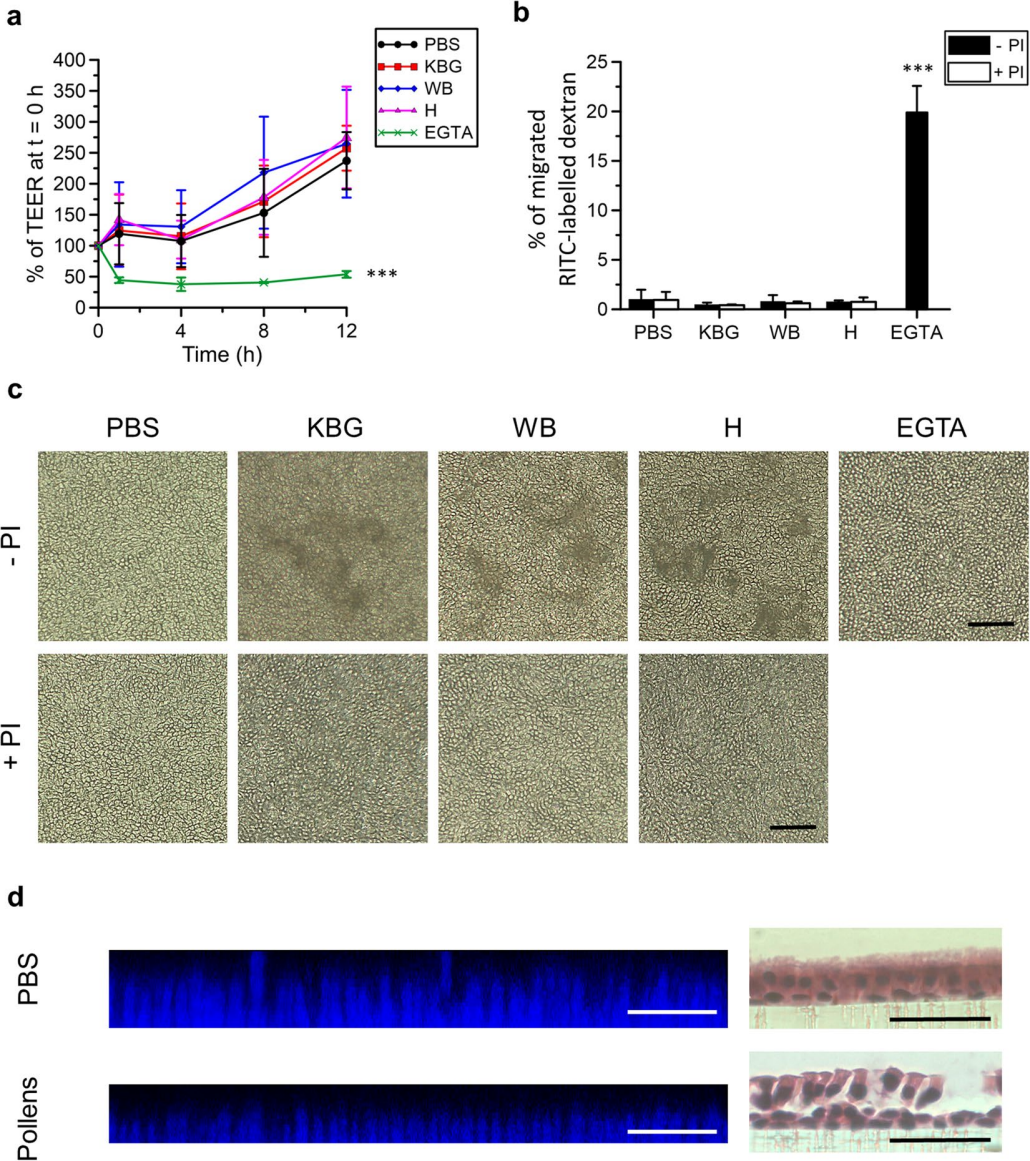
Pollen proteases selectively and irreversibly alter intercellular junctions of columnar primary equine respiratory epithelial cells (EREC). Polarized EREC were treated with PBS (control), pollen diffusates (Kentucky bluegrass or KBG, white birch or WB and hazel or H) or EGTA, supplemented with or without protease inhibitors (PI). (**a**) The transepithelial electrical resistance of EREC was measured over time and was normalized to time point 0 h. (**b**) Percentage of RITC-labelled dextran 70S in the basolateral chamber solution over that in the apical chamber solution. RITC-labelled dextran 70S was added 2 h after the initiation of the treatment and incubated for another 10 h (right panel). Three independent experiments were performed and the data are represented as means ± SD. Asterisks indicate significant differences (***P < 0.001). (**c**) Representative light microscopy pictures of EREC. Note the desquamating cells in EREC pretreated with pollen diffusates without PI (KBG, WB and H - PI). The scale bar measures 50 µm. (**d**) Cross-section of the EREC layer reconstructed from 20 consecutive confocal Z-stack images (left) or haematoxylin-eosin-stained paraffin coupes of the EREC layer (right) following treatment with PBS (control, upper panels) or pollens (lower panels). Cell nuclei are shown in blue (left) or purple (right) and the scale bar measures 50 µm.

ICJ integrity of EREC was examined by measuring both the transepithelial electrical resistance (TEER) and transepithelial permeability to RITC-labelled dextran. EREC reached a steady TEER of ~500–700 Ω × cm^−2^ after 5–7 days of incubation at the air-liquid interface in a transwell cell culture system. Starting from 30 min post addition, the TEER significantly dropped to baseline levels after treatment with EGTA (positive control), but not after treatment with PBS or with different pollen diffusates (Fig. 4a). In contrast, the TEER increased in a time-dependent manner and reached values that were up to 2.5-fold higher at 12 h after exposure than at 0 h in both PBS- and pollen diffusate-treated EREC. The TEER of additional EREC that did not receive any treatment, i.e. had air-filled apical compartments, remained stable throughout the experiment (data not shown). TEER values did not significantly differ among PBS- and pollen diffusate-treated EREC. The pattern of TEER values over time was similar in EREC treated with pollen diffusates supplemented with PI, as in EREC treated with pollen diffusates without PI (data not shown). Similarly, the transepithelial permeability, as assessed by the percentage of migrated RITC-labelled dextran across the EREC layer and shown in Fig. 4b, did not significantly differ between EREC exposed for 12 h to different pollen diffusates and control-treated EREC. Chelation of extracellular Ca^2+^ with EGTA, however, was able to significantly increase the percentage of migrated RITC-labelled dextran across the EREC up to 20%. Treatment with all three pollen diffusates, but not with PBS, EGTA or after supplementation of PI, induced cellular desquamation. Illustrative light microscopy pictures are given in Fig. 4c. The loss of columnar epithelial cells upon pollen diffusate pretreatment was also apparent on the 3D reconstruction of 20 consecutive Z-stack confocal images of the EREC layer, shown in the left panels of Fig. 4d and on haematoxylin-eosin-stained images of the EREC layer, shown in the right panels of Fig. 4d. Viability of the EREC was not significantly affected upon treatment with pollen diffusates with or without PI, as determined by EMA-staining (Fig. S2b).

Together, these results show that integrity and anchorage of the columnar respiratory epithelial cell layer is selectively disrupted by pollen proteases, while the basal cell layer resists their damaging effect.

### Pollen proteases predispose respiratory epithelial cells for efficient EHV1 infection

#### Respiratory mucosal explants

**Figure 5:**
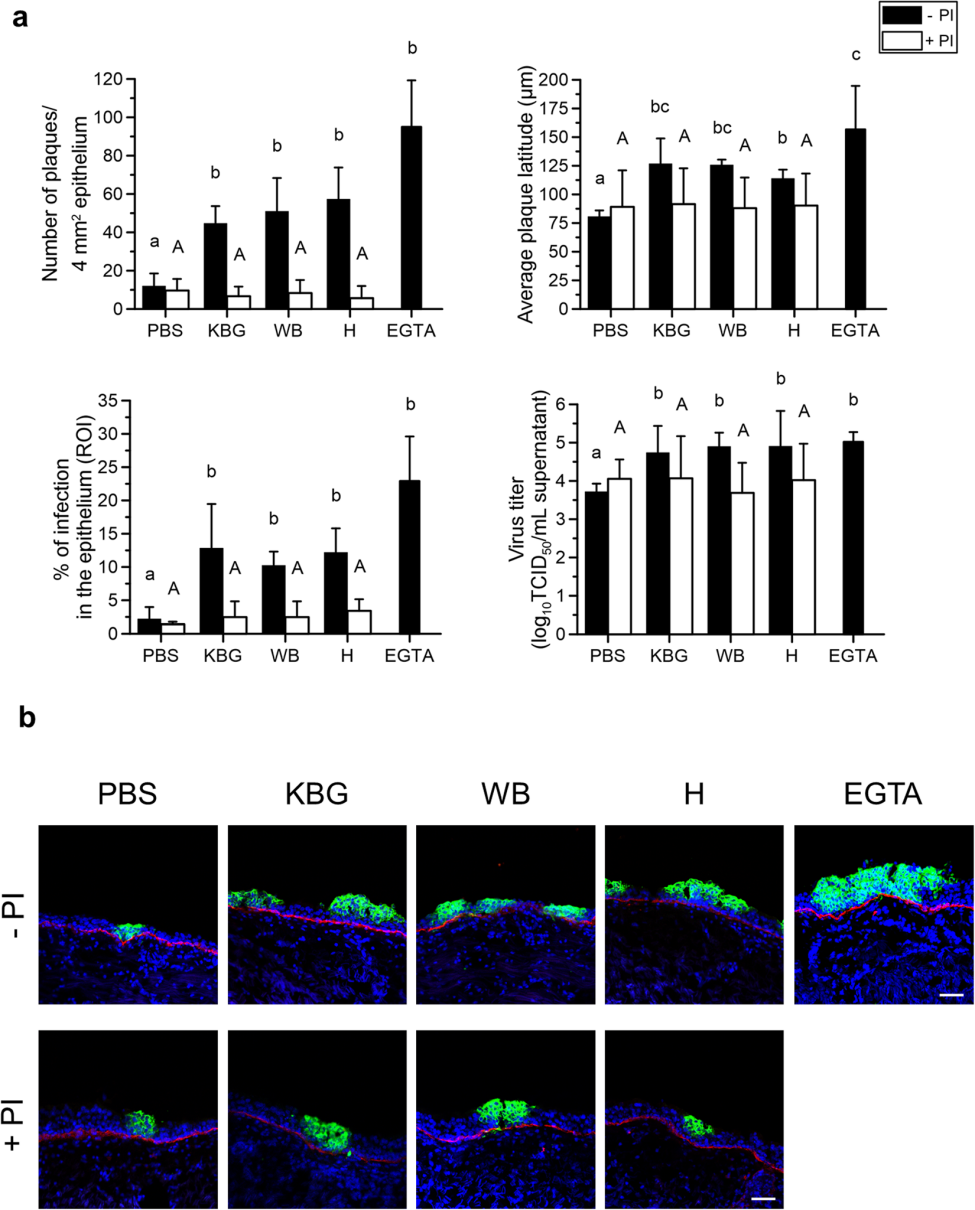
EHV1 infection of respiratory mucosal explants after pretreatment with PBS (control), pollen diffusates (Kentucky bluegrass or KBG, white birch or WB and hazel or H) or EGTA, supplemented with or without protease inhibitors (PI). Explants were frozen 24 hpi and cryosections were stained for late viral antigens. (**a**) The total number of plaques was counted on 50 consecutive cryosections (upper left panel), the average plaque latitude was calculated based on a maximum of 10 individual plaques (upper right panel), the percentage of EHV1 infection in the epithelium was analysed on 5 random cryosections (lower left panel) and the virus titer (lower right panel) was determined in supernatant on RK13 cells. Experiments were performed on explants from 3 individual horses and data are represented as means + SD. Different lower case letters indicate significant (P < 0.05) differences in treatments without PI, different upper case letters indicate significant (P < 0.05) differences in treatments with PI. (**b**) Representative confocal images of EHV1 plaques (green) in respiratory mucosal explants. The basement membrane is shown in red. Cell nuclei were counterstained with Hoechst 33342 (blue). The scale bar represents 50 μm.

Number of plaques: As shown in Fig. 5a, upper left panel, the number of plaques per 50 cryosections significantly (P < 0.05) increased from 12 ± 6 in PBS-pretreated explants (negative control) to 45 ± 9 (KBG), 51 ± 17 (WB) and 57 ± 16 (H) in pollen diffusate-pretreated explants. The number of plaques was significantly (P < 0.05) higher after EGTA pretreatment (positive control), compared to PBS pretreatment and did not significantly differ from the number of plaques in the pollen diffusate-pretreated explants. Inhibition of the proteases in the pollen diffusates with PI completely prevented the increase in subsequent EHV1 infection, as the number of plaques following pollen diffusate with PI pretreatment (6 ± 5 for KBG, 8 ± 7 for WB and 6 ± 6 for H) did not significantly differ from PBS pretreatment (with or without PI; 10 ± 6 and 12 ± 6, respectively).

Plaque latitude: The plaque latitude gives an indication about the ease of viral spread in the explant epithelium and is shown in Fig. 5a, upper right panel. The average latitude of EHV1 plaques increased significantly from 81 ± 5 μm in PBS-pretreated explants to 127 ± 22 μm (KBG), 126 ± 5 μm (WB) and 114 ± 8 µm (H) after pollen diffusate pretreatment. Similarly, the average EHV1 plaque latitude was higher after EGTA treatment (157 ± 38 μm), compared to control. The average EHV1 plaque latitude in pollen diffusate with PI-pretreated explants (92 ± 31 µm for KBG, 88 ± 27 µm for WB and 90 ± 28 µm for H) was similar to PBS-pretreated explants (with or without PI; 89 ± 32 µm and 81 ± 5 µm, respectively).

Percentage of infection in the epithelium: In order to obtain a general overview of EHV1 infection in explants, the percentage of infection in the epithelium (i.e. ROI) was calculated and is illustrated in Fig. 5a, lower left panel. In PBS-pretreated explants, 2.20 ± 1.78% of the epithelium was infected by EHV1. Pretreatment of the explants with pollen diffusates led to a significant (P < 0.05) increase of this percentage to 12.83 ± 6.63% (KBG), 10.24 ± 2.07% (WB) and 12.19 ± 3.61% (H). Up to 22.90 ± 6.70% of the epithelium got infected by EHV1 after pretreatment with EGTA. A similar trend as in the number of plaques was observed for the percentage of infection in the epithelium of explants pretreated with PBS or pollen diffusates supplemented with PI. Here, 1.41 ± 0.37% (PBS), 2.46 ± 2.38% (KBG), 3.38 ± 1.01% (WB) and 3.41 ± 1.74% (H) of the epithelium was infected with EHV1.

Virus titer: More efficient virus replication in the epithelium most likely results in the production of more extracellular virus particles. Indeed, virus titration (Fig. 5a, lower right panel) confirmed this hypothesis showing that the supernatant of all pollen diffusate-pretreated explants contained on average a 1 to 1.5 log_10_ higher titer (4.74 ± 0.70 for KBG, 4.90 ± 0.36 for WB and 4.91 ± 0.92 for H) than control-pretreated explants (3.84 ± 0.2). EGTA-pretreated explant supernatant was on average 1.5 log_10_ higher (5.02 ± 0.26) than control supernatant. Again, preventing protease activities by supplementing PI to the pollen diffusates resulted in a similar virus titer (4.06 ± 1.1 for KBG, 3.69 ± 0.78 for WB and 4.02 ± 0.96 for H), compared to control (3.72 ± 0.21).

Representative confocal images of EHV1 plaques in different pretreated respiratory mucosal explants are shown in Fig. 5b.

#### EREC

**Figure 6:**
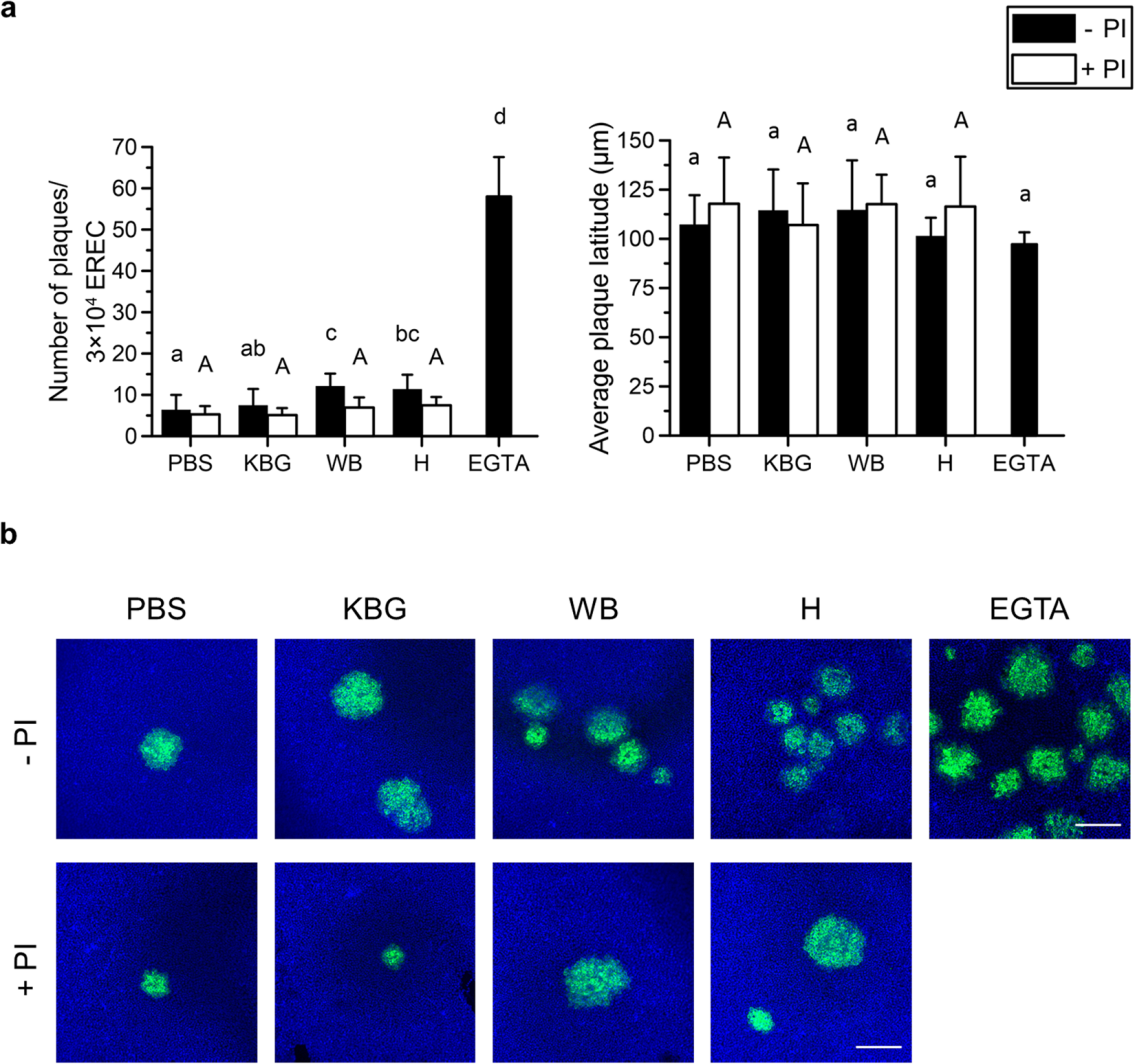
EHV1 infection of equine respiratory epithelial cells (EREC) after pretreatment with PBS (control), pollen diffusates (Kentucky bluegrass or KBG, white birch or WB and hazel or H) or EGTA, supplemented with or without protease inhibitors (PI). Cells were fixed 10 hpi and stained for immediate early protein (IEP). (**a**) The total number of plaques was counted in five different fields of approximately 3 × 10^4^ cells for each condition (left). Average plaque latitudes were measured on 10 individual plaques (right). Experiments were performed in triplicate on primary EREC of 3 different horses. Data are represented as means + SD and different lower case letters indicate significant (P < 0.05) differences in treatments without PI, different upper case letters indicate significant (P < 0.05) differences in treatments with PI. (**b**) Representative confocal images of EHV1 IEP-positive plaques (green) in EREC monolayers, nuclei were detected with Hoechst 33342 (blue). The scale bar represents 75 μm.

Number of plaques: On 3 × 10^4^ EREC, we counted an average of 6 ± 4 EHV1 plaques following PBS pretreatment of the cells. Disruption of columnar cell ICJ with proteases of KBG, WB and H increased the number of EHV1 plaques in 3 × 10^4^ EREC to 8 ± 4, 12 ± 3 and 11 ± 4, respectively. Complete disruption of epithelial integrity with EGTA completely overcame the restriction in EHV1 infection upon apical inoculation, resulting in an average of 58 ± 10 EHV1 plaques per 3 × 10^4^ EREC (Fig. 6a, left panel). Addition of PI to the pollen diffusates during EREC pretreatment inhibited the increase in EHV1 infectivity.

Plaque latitude: No significant difference in EHV1 plaque latitude was found between different pretreatments (Fig. 6a, right panel).

Representative confocal images of EHV1 plaques in different pretreated EREC are shown in Fig. 6b.

Taken together, the partial disruption of respiratory epithelial integrity by pollen proteases was sufficient to enhance subsequent EHV1 infection, but not to the same level as after a complete disruption of epithelial integrity with EGTA.

## Discussion

In today’s modern society, the number of patients developing asthma and allergies is dramatically increasing^33,34^. Our respiratory tract is constantly exposed to respirable hazards from the industrialized world, ranging from moulds and other airborne pathogens to air pollutants, dust and pollens. All of these substances are potentially involved in the initiation or progression of allergic diseases, but pollens are among the gold standards^3,35,36,37^. Upon hydration either outside or inside the respiratory tract, pollens release a repertoire of proteins, including allergens and proteases^24,38^. All these compounds are of key importance in the onset of pollen allergy and it is generally believed that pollen proteases affect the epithelial barrier, thereby facilitating the para-cellular transport of pollen allergens^6–8,39^. However, the exact impact of pollen proteases on the respiratory epithelium remains to be fully characterized, as representative human models are sparse. Interestingly, Bullone and Lavoie^19^ pointed out that the horse animal model is well suited to study human respiratory allergies. Similarly, horses commonly suffer from pollen allergies, which is known as summer pasture-associated recurrent airway obstruction (SP-RAO)^18^. Moreover, human asthma and equine RAO share common clinical presentation and also important immunological and tissue remodelling features^19^. Further, we hypothesized that pollen-induced loss of respiratory epithelial barrier function might facilitate the invasion of the alphaherpesvirus EHV1.

Consistent with previous studies, we observed that PBS-submerged pollens release high molecular weight proteases with major metalloproteinase and serine peptidase activities^6,7,9,24^. These results were corroborated by our proteomics-derived data of the white birch (WB) pollen diffusate. Indeed, we mainly identified serine proteases of the subtilase family in all three WB proteolytic lanes, besides the metalloproteinase meiotic prophase aminopeptidase 1 in the 100–150 kDa lane. The identified subtilases presumably occur in multiple forms, as some of them (e.g. Bpev01.c0015.g0096.m0001 or subtilisin-like serine protease 1.7) were derived from different proteolytic lanes following non-denaturing SDS PAGE. Multiple other proteases were presumably present in the WB proteolytic lanes, as also EDTA and E-64 could efficiently block enzymatic activity on zymograms and the majority of MS/MS spectra could not be assigned to specific proteins by means of proteomics. For instance, our zymography results showed the presence of an additional major metalloproteinase lacking serine protease activities in the >250 kDa band and a cysteine protease in the 70 kDa band of WB, but proteomics did not (yet) allow us to identify these proteases. Future knowledge on the complete protein database of WB should eventually unravel the identities of these putative proteases.

Previous studies using human continuous cell lines found that pollen proteases affect epithelial integrity by destroying major constituents of the intercellular junctions (ICJ), namely tight junction proteins (occludin, zonula occludens-1 protein, claudin-1) and adherent junction proteins (E-cadherin)^6,7,9^. Similarly, we showed that pollen proteases disrupt ICJ and anchorage of respiratory epithelial cells in both *ex vivo* equine respiratory mucosal explants and primary EREC. In addition, we analysed the disruption of specific intercellular junction proteins by means of immunofluorescence staining (e.g. occludin and E-cadherin). However, none of the commercially available antibodies cross-reacted with the equine forms of the intercellular junction proteins. Remarkably, we found that only the ICJ of columnar epithelial cells were affected by pollen proteases. This phenomenon was strikingly different from that observed after addition of the calcium-depleting agent EGTA, which rapidly caused splitting of both basal and columnar cell intercellular junctions^29^. As a result, the damage caused by pollens to the ICJ must have been independent of calcium concentration. First, the degradation of ICJ might be a direct consequence of proteolytic actions. The exposed domains of basal cell transmembrane proteins might be less accessible to exogenous proteases, compared to those of columnar cells. A more tighter sealing in between basal cells would not be surprising, as loss of these cells would have detrimental effects on the epithelium. Indeed, basal cells not only function as progenitor cells, but also play a major role in fixation of the complete respiratory epithelium by expressing hemidesmosomal complexes^40,41^. Second, activation of a protease-activated receptor (PAR) at the surface of epithelial cells could also induce disruption of epithelial integrity^42^. PAR are G protein-coupled receptors that are activated after proteolytic cleavage of their N-terminus^43^. It was proposed by Vinhas, *et al*.^6^ that disruption of the epithelial barrier by pollen proteases could occur via the activation of a PAR. Allergenic products of mites, moulds and the Japanese Cedar pollens have already been shown to activate PAR2, thereby initiating the disassembly of ICJ^44–46^. Interestingly, PAR1 and 2 are exclusively expressed by columnar epithelial cells of the human respiratory tract and/or skin^47,48^. However, due to the lack of commercially available antibodies against equine PAR, the visualisation of these receptors in the equine respiratory tract was not possible. Further support for a potential contribution of PAR2 in the pollen-induced loss of epithelial integrity comes from our zymography results, as almost all proteolytic activities of the pollen diffusates were blocked with AEBSF. In addition, we mainly identified serine proteases of the subtilase family in the WB pollen diffusate using proteomics. Notably, PAR2 is selectively activated by serine proteases, through cleavage of its N-terminus^49^. Future studies will determine the effect of equine PAR2 agonists on respiratory epithelial cell integrity.

We additionally identified a metalloproteinase ‘meiotic prophase aminopeptidase 1’ in the WB pollen diffusate, together with a plethora of glycosidases and lipases. As described for matrix metalloproteinase 9, meiotic prophase aminopeptidase 1 might also alter airway epithelial integrity^50^. Based on these findings, it seems reasonable to propose that pollen-derived glycosidases clear passage through the mucus blanket by destroying the mucoprotein network. This strategy not only allows for the delivery of pollen proteases and allergens, but also airborne pathogens to the respiratory epithelium. Interestingly, our respiratory mucosal explant model is also able to maintain normal mucus production throughout the incubation period, as shown in the confocal images of Fig. S3. This finding further adds to the statement that the respiratory mucosal explant model represents the *in vivo* environment very well, rendering it ideal to study the impact of pollens on the respiratory tract.

Pollen-derived proteases clearly caused irreversible damage to the respiratory epithelium. Twenty-four hours after normalizing the medium, merely a thin layer of basal cells persisted in the explants upon treatment with pollen proteases. The normal ciliated appearance of the respiratory epithelium disappeared due to loss of desquamated and apoptotic epithelial cells. *In vivo*, this reduced mucociliary clearance might even further promote the damaging effect of pollen substances, as the induced stasis might further concentrate them in one place. It would be interesting to study the regeneration capacity and duration of the respiratory epithelium. However, viability of respiratory mucosal explants rapidly decreases following 4 days of incubation^16^. *In vivo* studies would be better suitable to study the latter regeneration mechanisms, although they raise ethical questions.

Cellular desquamation was also observed in the EREC upon exposure to pollen diffusates, which is in line with previous studies^9,51^. However, in contrast with studies using continuous cell lines, we did not observe a decrease in transepithelial electrical resistance or an increase in transepithelial permeability across the EREC upon apical exposure to the pollen diffusates^6,52^. This corroborates our observations in *ex vivo* respiratory mucosal explants, where integrity of the basal cells was not affected upon treatment with pollen diffusates. Upon loss of their neighbouring epithelial cells, the basal cells may have rapidly flatted out and thereby covered the underlying surface, as they would do *in vivo*^53^. Indeed, haematoxylin-eosin-stained paraffin coupes of the EREC layer confirmed that basal cells resisted pollen protease treatment, while covering the transwell membrane. Surprisingly, coverage of the apical EREC surface with either PBS or pollen diffusates led to an increase of their TEER. As these results are consistent with those obtained from primary human bronchial epithelial cells after exposure to grass pollen extracts, we want to emphasize the importance of using appropriate representative models^10^. Here, the increase in epithelial integrity might have acted as a self-defence mechanism in a harmful environment^54^. Indeed, respiratory epithelial cells generally thrive well at the air-liquid interface, but fail to maintain their differentiation level after full immersion in medium^55–57^.

We previously showed that upon destruction of epithelial integrity, infection of the respiratory epithelium with the alphaherpesvirus equine herpesvirus type 1 (EHV1) was greatly enhanced^29^. As this ancestral alphaherpesvirus targets a receptor located at the basolateral surface of epithelial cells, primary infections are limited and most often occur subclinically. Here, we demonstrated that the respiratory epithelium is more easily infected by EHV1 upon disruption of the ICJ by pollen proteases. Nonetheless, the increase in subsequent EHV1 infection was not to the same degree as following a complete destruction of epithelial integrity with EGTA. Indeed, the basal cells remained able to seal the epithelium upon exposure to pollen proteases, avoiding unlimited passage of virus particles. Nonetheless, the basolateral surface of (semi-)detaching columnar cells was still exposed to virus particles, enabling efficient infection. Especially in the more representative respiratory mucosal explants, EHV1 infection was predominantly enhanced upon pollen protease pretreatment, which was less obvious in EREC. Although the EREC, grown in optimal conditions, differentiate into a pseudo-stratified respiratory epithelium, they presumably cannot fully mimic the *in vivo*-differentiated respiratory epithelium of mucosal explants^12^.

Our results are corroborated by the seasonally-observed EHV1-associated symptoms (i.e. respiratory disease, central nervous system disorders and abortion), as these occur most often during late winter and spring, when pollen concentrations in the outdoor air peak^58–60^. Indeed, in Europe, hazel is the first to shed pollens in the air (December–April), followed by birch (March–April) and grasses (May–June)^4^. Nonetheless, other factors such as crowding of horses during winter and the seasonal breeding cycle of horses also contribute to these seasonally-observed symptoms.

Vice versa, the enhanced replication of (alpha)herpesviruses in the respiratory epithelium might further promote the detrimental effects of pollens. First, the viral-induced destruction of epithelial cells leads to impaired mucociliary clearance, which may further concentrate pollen substances in one place^61^. Furthermore, epithelial damage will facilitate the delivery of these pollen substances to the underlying cells of the immune system to promote airway sensitization^3,5,62^. Next, alphaherpesviruses might selectively activate Th2 cells or suppress T regulatory cells in certain individuals, which are both of key importance in the development of allergies. Indeed, Th2 responses have already been shown to exacerbate HSV1-induced keratitis in mice^63^. However, further studies using appropriate host-specific models are required to uncover the role of alphaherpesviruses in Th2 cell priming.

Besides alphaherpesviruses, multiple other airborne pathogens might co-migrate along with pollen substances and benefit from this barrier-dysfunction to enhance infection. For example, adenoviruses and reoviruses likewise preferentially infect the basolateral surface of respiratory epithelial cells^64,65^. In addition, damage to the ciliated epithelial cells by pollen proteases might favour subsequent attachment of and colonisation by bacteria (e.g. *Streptococcus* species)^66,67^.

Together, we shed new light on the detrimental effects of pollens on the respiratory epithelium and their role in pathogen invasion. More precisely, we found that pollen proteases selectively and irreversibly disrupt integrity and anchorage of columnar respiratory epithelial cells, while the indispensable basal cells are resistant and succeed in maintaining the epithelial barrier. Upon exposure to pollen proteases, the flatted basal cells can partly prevent the alphaherpesvirus EHV1 from reaching its binding receptor, located basolaterally in the epithelium. However, they cannot fully prevent an increase in EHV1 infection of the respiratory epithelium, as the virus can still reach the basolateral surface of semi-detaching epithelial cells. Finally, we hypothesize that a concurrent alphaherpesvirus invasion of the respiratory mucosa might further drive the onset and persistence of allergies through mechanisms yet to be elucidated.

## Methods

### Preparation of pollen diffusates

Pollens of Kentucky bluegrass (KBG; *Poa pratensis*), white birch (WB; *Betula pendula*) and hazel (H; *Corylus americana*) were purchased from Stallergeens Greer (Cambridge, MA, USA). Pollens were shaken at a concentration of 100 mg/mL in phosphate-buffered saline (PBS) for 2 h at 4 °C prior to centrifugation at 10,000 g for 10 min at 4 °C. The supernatant was harvested and filtered through 0.22 µm pore size filters (VWR International, Radnor, PA, USA). Protein concentration in the pollen diffusates was determined using the Bradford assay (ThermoFisher Scientific, Waltham, MA, USA) and a Nanodrop^®^ spectrophotometer, following the manufacturer’s instructions. All pollen diffusates were diluted with PBS to a final concentration of 5 mg/mL and either used immediately or frozen in aliquots at −20 °C.

### Gel electrophoresis and Zymography

To assess protein and proteolytic profiles of the pollen diffusates, SDS-polyacrylamide gel electrophoresis (SDS-PAGE) followed by either Coomassie blue staining alone or zymography and Coomassie blue staining, respectively, was performed. Proteolytic activities were characterized by using different protease inhibitors (PI) in zymography. Full details are given in the supplementary information (SI).

### Proteomics

#### Sample preparation

White birch protein bands with corresponding enzymatic activity, as assessed by zymography, were excised from the gel following SDS-PAGE, washed two times in H_2_O and frozen at −20 °C until further processing. Gel bands were digested overnight at 37 °C using trypsin (Promega). Peptides eluted from every gel band were dried completely in a vacuum concentrator and re-dissolved in 20 µl loading solvent A (0.1% TFA in water/acetonitrile [98:2, v/v]) for subsequent LC-MS/MS analysis. Full details are given in SI.

#### LC-MS/MS and Data Analysis

LC-MS/MS analysis was performed on an Ultimate 3000 RSLCnano system in-line connected to an LTQ Orbitrap Elite mass spectrometer (ThermoFisher Scientific). Data analysis was performed with MaxQuant (version 1.6.1.0) using the Andromeda search engine with default search settings including a false discovery rate set at 1% on both the peptide and protein level. Spectra from all three gel bands were searched together against a database of *Betula pendula* protein sequences reported by Salojärvi, *et al*.^32^ (containing 29,919 protein sequences downloaded from https://genomevolution.org on March 27 2018). A detailed description of the LC-MS/MS and data analysis is given in SI.

### Isolation and cultivation of respiratory mucosal explants and primary equine respiratory epithelial cells (EREC)

The tracheae from different healthy horses were collected at the slaughterhouse. Tracheal mucosal explants and primary equine respiratory epithelial cells (EREC) were isolated and cultured as previously described^12,16,29^.

### Pollen diffusate treatment of respiratory mucosal explants and EREC

#### Respiratory mucosal explants

Explants were cultured 24 h for adaptation before thoroughly washing and embedding them in agarose diluted in 2X MEM, to mimic *in vivo* conditions, as previously published^29,68^. Next, the apical surface of the epithelium was exposed for 12 h at 37 °C to the pollen diffusates supplemented with or without the protease inhibitor mixture (PI) containing 500 µM AEBSF, 15 µM E-64 and 2.5 µM EDTA. As negative control, PBS with or without PI was used. One hour treatment with EGTA was included as positive control^29^. Explants were removed from the agarose and washed three times in PBS and fixed in a phosphate-buffered 3.5% formaldehyde solution for 24 h, either immediately after the last wash or after an additional 24 h incubation on metal gauzes. Explants were then stored into 70% alcohol until further processing. To guarantee a sufficient viability (>90%) of the explants after treatment with different pollen diffusates, an ‘*In Situ* Cell Death Detection Kit’ (Roche Diagnostics Corporation, Basel, Switzerland), based on terminal deoxynucleotidyl transferase dUTP nick end-labelling (TUNEL), was used.

#### EREC

Cells were grown to confluency and the transepithelial electrical resistance (TEER) was measured daily until a steady TEER of ~500–700 Ω × cm^−2^ was reached. The pollen diffusates with or without the above-described PI were added to the apical surface of the cells. EGTA was used as positive control and PBS with or without PI was used as negative control. Cells were incubated for 12 h, while epithelial cell integrity was assessed, as described below. Viability of the cells was assessed by ethidium monoazide bromide (EMA)-staining, ensuring that the treatment with pollen diffusates did not cause a significant cell loss.

### Assessment of epithelial cell integrity

#### Respiratory mucosal explants

Integrity of the intercellular junctions was verified by examining the intercellular space in haematoxylin-eosin-stained paraffin coupes, as previously described^29^. The percentage of intercellular space in and thickness of the epithelium was measured using ImageJ software (ImageJ, U.S. National Institutes of Health, Bethesda, MD, USA).

#### EREC

To assess epithelial integrity of the EREC, both the transepithelial electrical resistance (TEER) and the migration of rhodamine B isothiocyanate (RITC)-labelled dextran 70 S (Sigma-Aldrich) across the epithelium were determined. Full details are given in SI.

### Viral infection assays

#### Virus

A Belgian EHV1 isolate (03P37) was used in this study and originates from the blood taken of a paralytic horse during an outbreak in 2003^69^. The virus was propagated on rabbit kidney (RK13) cells and used at the 6^th^ passage.

#### Respiratory mucosal explants

Explants were cultured at the air-liquid interface for 24 h, prior to extensive washing and embedment in agarose. Next, explants were exposed to the pollen diffusates or PBS with or without PI (negative control) for 12 h or to EGTA (positive control) for 1 h, as described above. Following a washing step, the apical surface of the epithelium was inoculated with 10^6.5^ TCID_50_ of the 03P37 EHV1 strain for 1 h at 37 °C. Explants were removed from the agarose and washed 3 times in PBS to remove non-adherent virus particles. Finally, explants were placed back onto their gauzes and serum-free medium was added. Twenty-four hpi, explants were placed in methylcellulose-filled plastic tubes and frozen at −80 °C until further processing.

#### EREC

EREC were grown to full differentiation in a transwell cell culture system prior to treatment with the pollen diffusates with or without PI, EGTA (positive control) or PBS with or without PI (negative control). Following a washing step, cells were exposed for 1 h to 100 μL EHV1 03P37 strain (MOI of 1) at the apical surface. Non-adsorbed virus particles were removed by washing the EREC three times with DMEM/F12. Fresh EREC medium was added to the platewells and cells were further incubated at the air-liquid interface. Ten hours post inoculation, cells were fixed in methanol for 20 min at −20 °C and stored dry at −20 °C until further processing.

### Immunofluorescence staining and confocal microscopy

#### Respiratory mucosal explants

Explants were embedded in methylcellulose and snap-frozen for subsequent cryosectioning.

Cryosections were immunofluorescently stained to label late viral glycoproteins and the basement membrane. Further details are given in SI.

#### EREC

Immunofluorescent staining to visualize EHV1 immediate early protein (IEP) and cell nuclei was performed directly in the transwells. Further information is given in SI.

### Virus titration

Twenty-four hours after inoculation, explant supernatant was collected and stored at −80 °C until titration. EHV1 titrations were conducted on RK13 cells, which were incubated at 37 °C for 7 days. Titers were expressed as TCID_50_.

### Statistical analyses

Significant differences (P < 0.05) between different treatments were identified by one-way analysis of variances (ANOVA) followed by Tukey’s post-hoc test. If homoscedasticity of the variables was not met as assessed by Levene’s test, the data were log-transformed prior to ANOVA. Normality of the residuals was verified by the use of the Shapiro-Wilk test. If the variables remained heteroscedastic or normality was not met after log-transformation, a Kruskall-Wallis test, followed by a Mann-Whitney post-hoc test was performed. All analyses were conducted in IBM SPSS Statistics for Windows, version 25.0 (IBM Corp, Armonck, NY, USA).

## Supplementary information

Supplementary Information

## Data Availability

The datasets generated and/or analysed during the current study are available from the corresponding author on reasonable request.
