# Supplementary material for: Pollens destroy respiratory epithelial cell anchors and drive alphaherpesvirus infection
Source: Sci Rep. 2019 Mar 18;9:4787. doi: 10.1038/s41598-019-41305-y (PMC6423322; doi:10.1038/s41598-019-41305-y)
Supplement: Supplementary file 1 — Supplementary Information [file 41598_2019_41305_MOESM1_ESM.pdf]

Supplementary Information

Pollens destroy respiratory epithelial cell anchors and drive alphaherpesvirus infection

Jolien Van Cleemput, Katrien C.K. Poelaert, Kathlyn Laval, Francis Impens, Wim Van den Broeck, Kris Gevaert, Hans J. Nauwynck.

Supplementary Figures

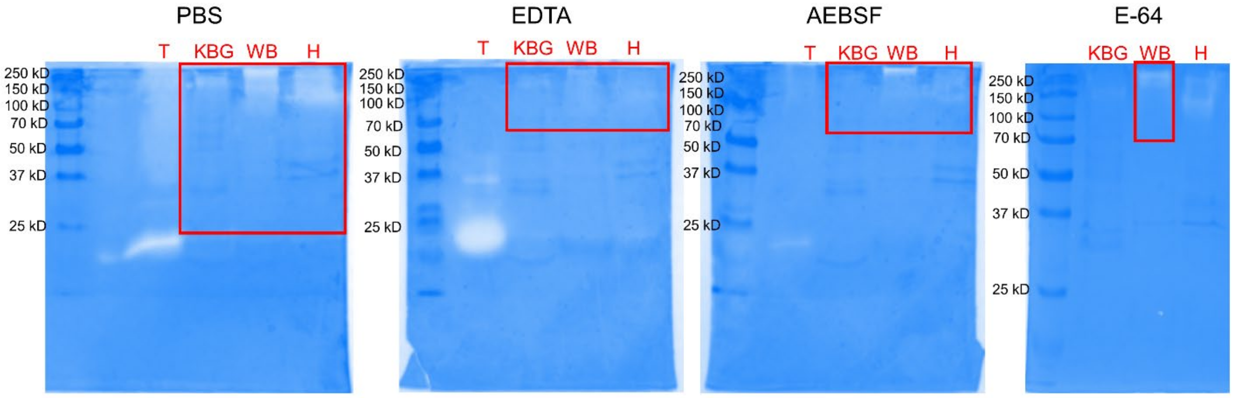

Figure S1. Corresponding complete zymograms of Figure 1.

Pollen diffusates of Kentucky bluegrass (KBG), white birch (WB) and hazel (H) contain proteolytically active compounds. Pollen diffusates were incubated with PBS or several protease inhibitors (2.5 µM EDTA, 500 µM AEBSF or 15 µM E-64) prior to and during zymography on a gelatin substrate. Trypsin (T) was included as positive control for zymography. Following overnight digestion at 37°C, Coomassie blue staining of the gelatin gels was performed. Proteolytic bands appear as white zones in the Coomassie blue-stained gelatin gels. Red boxes were selected and magnified in Figure 1.

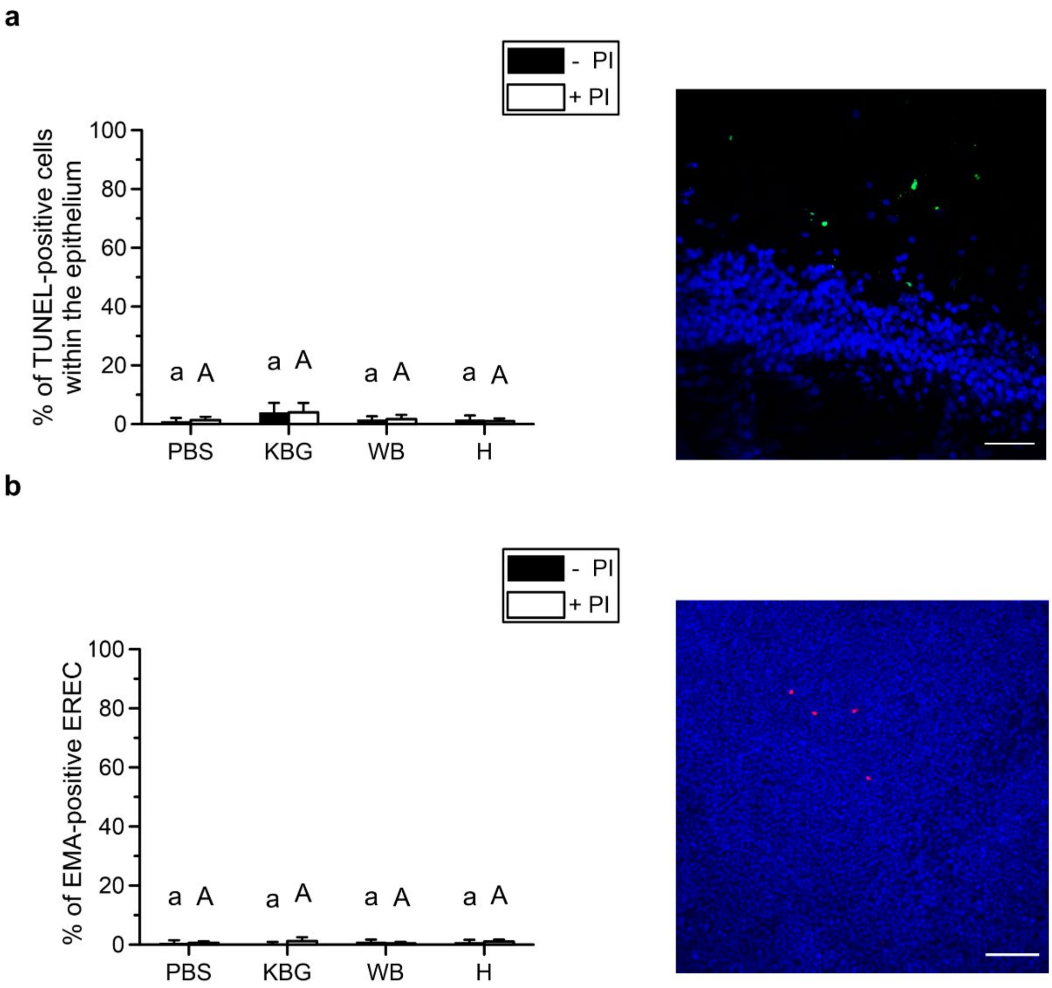

Figure S2. Cell viability in explants (a) and EREC (b) upon pollen diffusate treatment

Cell viability of the cells within the epithelium was not affected upon treatment with pollen diffusates of Kentucky bluegrass (KBG), white birch (WB) or hazel (H), supplemented with or without protease inhibitors (PI). (a) TUNEL staining data of 12 h pollen diffusate-treated respiratory mucosal explants. Experiments were performed on explants from 3 individual horses and data are represented as means + SD. The lower case letters indicate significant ($P<0.05$) differences in treatments without PI, different upper case letters indicate significant ($P<0.05$) differences in treatments with PI (left). Representative confocal image of pollen protease-induced apoptosis (shown in green) in detaching epithelial cells. Cell nuclei are shown in blue. The scale bar represents 50 µm (right). (b) EMA staining confirmed no significant ($P<0.05$) differences in viability of the EREC monolayer after different treatments. Three independent experiments were performed and data are represented as means + SD. The lower case letters indicate significant ($P<0.05$) differences in treatments without PI, different upper case letters indicate significant ($P<0.05$) differences in treatments with PI (left). Representative confocal image of pollen diffusate-treated EREC. Cell nuclei are shown in blue and a postive EMA-signal is shown in red. Scale bar measures 100 µm (right).

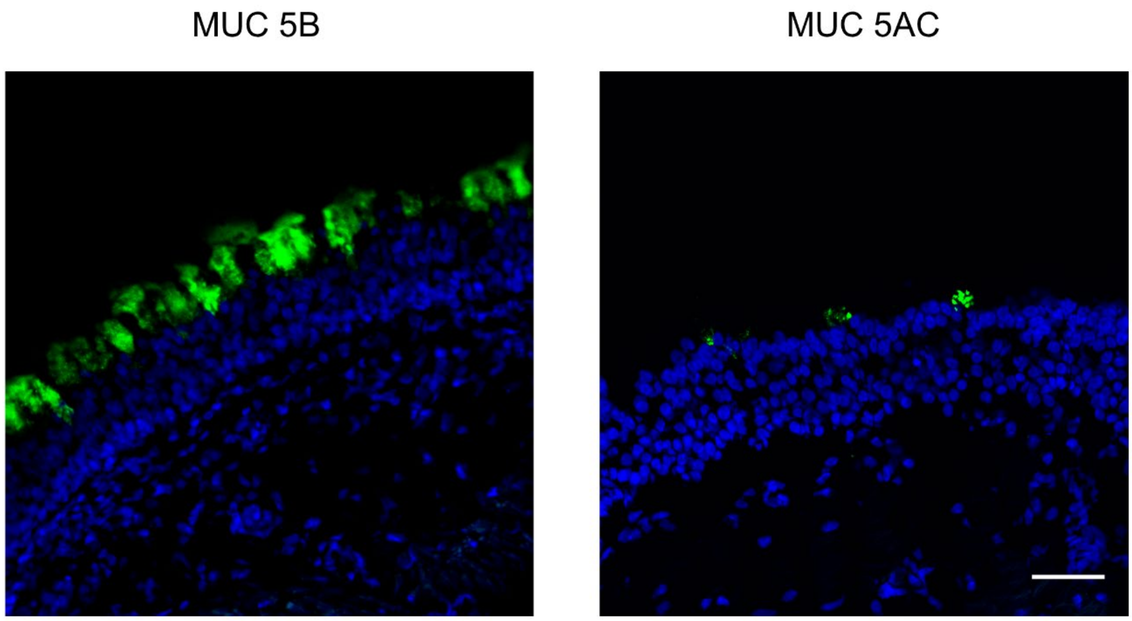

Figure S3. Respiratory mucosal explants produce mucins.

Immunofluorescence staining was performed to verify whether equine respiratory mucosal explants retain the capacity to produce mucins during culture. For this, equine tracheal mucosal explants were incubated for 24 h at 37°C, 5% CO_2, prior to snap-freezing. Sixteen µm thick sections were cut using a cryotome at -20°C and subsequently stained for mucin proteins (green) using a mouse monoclonal anti-MUC 5B antibody (clone 19-2E; Santa Cruz Biotechnology, Dallas, Texas, USA) or a mouse monoclonal anti-MUC 5AC antibody (clone CLH2; Santa Cruz Biotechnology), followed by incubation with a FITC®-labelled polyclonal goat anti-mouse IgG (ThermoFisher Scientific). Cell nuclei were counterstained in blue using Hoechst 33342.

Supplementary Methods

Gel electrophoresis and Zymography

Gel electrophoresis

Pollen diffusates were diluted 1:1 in Laemmli sample buffer for 20 min at room temperature (RT) and loaded onto 10% SDS-polyacrylamide gels . The gels were run at 100 V for 110 min using a Mini Protean Tetra apparatus (Bio-Rad, Hercules, CA, USA). To assess the protein profile of the pollen diffusates, gels were stained for 1 h with Coomassie blue R-250 (Imperial™ Protein Stain; ThermoFisher Scientific) and destained in H_2O overnight.

Zymography

Prior to gel electrophoresis, pollen diffusates were incubated with or without protease inhibitors for 2 h at 4°C under gentle agitation. The following protease inhibitors were used: (i) an irreversible serine protease inhibitor 4-benzenesulfonyl fluoride hydrochloride (AEBSF); 500 µM (Sigma-Aldrich, St. Louis, MO, USA), (ii) a metal chelator ethylene diamine tetra-acetic acid (EDTA); 2.5 µM (VWR International, Radnor, PA, USA), (iii) an irreversible cysteine protease inhibitor epoxide 64 (E-64); 15 µM (Sigma-Aldrich) and (iv) a reversible aspartic acid protease inhibitor pepstatin A; 10 µM (Sigma-Aldrich). Gels impregnated with 0.1% gelatine (from bovine skin; Sigma-Aldrich) were used and gel electrophoresis was performed as described above. Next, proteins were renatured by incubating the gels in 2.5% Triton-X 100 for 20 min at RT. Following a washing step in H_2O, the gels were transferred to a new tray containing development buffer (50 mM Tris-HCl pH 7.5, 200 mM NaCl, 5 mM $CaCl_2$, 1 µM $ZnCl_2$; Sigma-Aldrich) supplemented with or without the above-described protease inhibitors. The gels were incubated overnight at 37°C and stained using Coomassie blue R-250, as described above. A ChemiDocMP Imaging System (Bio-Rad) was used to take pictures of the Coomassie blue-stained gels. RGB-pictures were converted to 8-bit grey-scaled images using ImageJ. Finally, presence or absence of proteolytic bands was determined by plot-profiling the zymogram lanes in ImageJ.

Proteomics

Sample preparation

Gel bands were washed with 500 µl H_2O, incubated for 15 min with 500 µl water/acetonitrile (1:1, v/v) and incubated for 15 min with 500 µl 100% acetonitrile before they were dried completely in a vacuum concentrator. Next, 150 ng sequencing-grade trypsin (Promega) in 50 mM ammonium bicarbonate in water/acetonitrile (9:1, v/v) was added to the dried gel bands and proteins were digested overnight at 37°C. Peptides eluted from every gel band were dried

completely in a vacuum concentrator and re-dissolved in 20 µl loading solvent A (0.1% TFA in water/acetonitrile (98:2, v/v)) for LC-MS/MS analysis.

LC-MS/MS and Data Analysis

From each gel band, 2 µl of re-dissolved peptides was injected for LC-MS/MS analysis on an Ultimate 3000 RSLCnano system in-line connected to an LTQ Orbitrap Elite mass spectrometer (ThermoFisher Scientific). Trapping was performed at 10 µl/min for 4 minutes in loading solvent A on a 20 mm trapping column (made in-house, 100 µm internal diameter (I.D.), 5 µm beads, C18 Reprosil-HD, Dr. Maisch, Germany) and the sample was loaded on a 200 mm analytical column (made in-house, 75 µm I.D., 1.9 µm beads C18 Reprosil-HD, Dr. Maisch). Peptides were eluted by a non-linear increase from 2 to 56% MS solvent B (0.1% FA in water/acetonitrile (2:8, v/v)) over 25 min at a constant flow rate of 250 nl/min, followed by a 10 min wash reaching 99% MS solvent B and re-equilibration with MS solvent A (0.1% FA in water/acetonitrile (2:8, v/v)). The mass spectrometer was operated in data-dependent mode, automatically switching between MS and MS/MS acquisition for the 20 most abundant ion peaks per MS spectrum. Full-scan MS spectra (300-2000 m/z) were acquired at a resolution of 60,000 in the orbitrap analyzer after accumulation to a target value of 3,000,000. The 20 most intense ions above a threshold value of 500 were isolated for fragmentation by CID at a normalized collision energy of 35% in the linear ion trap (LTQ), after filling the trap at a target value of 5,000 for maximum 20 ms (rapid scan rate mode).

Data analysis was performed with MaxQuant (version 1.6.1.0) using the Andromeda search engine with default search settings including a false discovery rate set at 1% on both the peptide and protein level. Spectra from all three gel bands were searched together against a database of *Betula pendula* protein sequences reported by Salojärvi, et al.[1] (containing 29,919 protein sequences downloaded from https://genomevolution.org on March 27 2018). The mass tolerance for precursor and fragment ions was set to 4.5 and 20 ppm, respectively, during the main search. Enzyme specificity was set as C-terminal to arginine and lysine (trypsin), also allowing cleavage at arginine/lysine-proline bonds with a maximum of two missed cleavages. Oxidation of methionine residues (to sulfoxides), acetylation of protein N-termini and propionamide modification of cysteine residues were set as variable modifications. Identified proteins were reported from the proteinGroups.txt MaxQuant output files and putative proteases, glycosidases and lipases (Table 1) were manually selected based on the annotation of the orthologues Arabidopsis proteins provided by Salojärvi, et al.[1].

Assessment of epithelial integrity on equine respiratory epithelial cells (EREC)

To assess epithelial integrity of the EREC, both the trans-epithelial electrical resistance (TEER) and the migration of rhodamine B isothiocyanate (RITC)-labelled dextran 70S (Sigma-Aldrich) across the EREC layer were determined. The TEER was measured using an epithelial voltohmmeter (Millipore corporation, Bedford, MA, USA) and the net resistance was calculated by subtracting the background resistance and multiplying the resistance by the surface area of the membrane. Following 2 h of incubation with the pollen diffusates, 50 µL of 100 µM RITC-labelled dextran 70S was added to the apical compartment of the transwell for another 10 h. Next, 50 µL of the basolateral compartment was transferred to a 96-well plate, suitable for fluorescent measurement (Greiner). Plates were measured at 530/590 nm, using a Flouroskan Ascent FL (ThermoFisher Scientific) plate reader. Finally, epithelial permeability was calculated as the percentage of RITC-labelled dextran 70S in the basolateral chamber solution over that in the apical chamber solution.

Immunofluorescence staining and confocal microscopy

Respiratory mucosal explants

Sixteen µm thick cryosections were cut using a cryostat at -20°C and loaded onto 3-aminopropyltriethoxysilane-coated (Sigma-Aldrich) glass slides. Slides were then fixed in 4% paraformaldehyde for 15 min and subsequently permeabilized in 0.1% Triton-X 100 diluted in PBS. Non-specific binding sites were blocked by 15 min incubation with avidin and biotin (ThermoFisher Scientific) at 37°C. To label late viral glycoproteins, a polyclonal biotinylated horse anti-EHV1 was used for 1 h at 37°C[2], followed by incubation with streptavidin-FITC® (ThermoFisher Scientific) for 1 h at 37°C. The basement membrane of the tissues was stained with monoclonal mouse anti-collagen VII antibodies (Sigma-Aldrich), followed by secondary Texas Red®-labelled goat anti-mouse antibodies (ThermoFisher Scientific). Nuclei were detected by staining with Hoechst 33342 (ThermoFisher Scientific). Slides were mounted with glycerol-DABCO and analysed using a Leica (TCS SPE) confocal microscope. The total number of plaques was counted on 50 cryosections and plaque latitude was measured using the Leica confocal software package. Five cryosections per explant were completely photographed and the percentage of infection in the epithelium (i.e. region of interest or ROI) was determined using ImageJ software.

EREC

Antibodies were incubated directly in the transwells for 1 h at 37°C. Cells were first incubated with a 1:1,000 dilution of a polyclonal rabbit anti-IEP antibody, kindly provided by Dr. D. O'Callaghan, Louisiana State University, USA. The diluent used was PBS containing 10%

negative goat serum. This was followed by incubation with a goat anti-rabbit IgG FITC®-conjugated antibody (ThermoFisher Scientific). Nuclei were counterstained with Hoechst 33342 for 10 min at 37°C. Transwell membranes were excised from the culture inserts and mounted on glass slides using glycerol-DABCO. Slides were examined using a Leica confocal microscope. The total number of plaques was counted on 5 random fields of approximately 3×10^4 cells per insert. Plaque latitude was measured on 10 individual plaques using the Leica confocal software package.

Supplementary References

1 Salojärvi, J. *et al.* Genome sequencing and population genomic analyses provide insights into the adaptive landscape of silver birch. *Nature genetics* **49**, 904 (2017).

2 van der Meulen, K., Vercauteren, G., Nauwynck, H. & Pensaert, M. A local epidemic of equine herpesvirus 1-induced neurological disorders in Belgium. *Vlaams Diergeneeskundig Tijdschrift* **72**, 366-372 (2003).
